# Research on the Effect of Commercial Health Insurance Development on Economic Efficiency

**DOI:** 10.3390/ijerph20065178

**Published:** 2023-03-15

**Authors:** Tongpu Zhao, Ruiyun Wanyan, Lingyan Suo

**Affiliations:** 1 School of Insurance and Economics, University of International Business and Economics, Beijing 100029, China; 2 Institute of Chinese Financial Studies, Southwestern University of Finance and Economics, Chengdu 610074, China; 3 School of Economics, Peking University, Beijing 100871, China

**Keywords:** commercial health insurance, economic efficiency, index construction

## Abstract

In the process of comprehensively promoting the construction of a multi-level medical security system in China, it is very important to clarify the impact of commercial health insurance. In order to better promote the development of commercial health insurance, we explore the effect of commercial health insurance development on economic efficiency. Theoretical analysis shows that, in addition to providing health risk protection for residents, commercial health insurance can also promote the coordinated development of the health industry chain, promote risk reduction, accumulate capital, and contribute to high-quality economic development. Empirically, this study indexes a commercial health insurance development index that is more in line with China’s development reality. In addition, this study compiles the economic efficiency index from the three dimensions of economic development basis, social benefits and industrial changes. We measure the commercial health insurance development index and economic efficiency index in 31 regions from 2007 to 2019, and further econometric analysis is carried out on this basis. It is found that the development of commercial health insurance can promote economic efficiency, and this result is robust. Meanwhile, the impact of commercial health insurance on economic efficiency is restricted by the economic environment itself, and the more developed the economy is, the more obvious this effect will be. Therefore, the development of commercial health insurance will significantly benefit the construction of China’s multi-level medical security system and promote regional economic efficiency.

## 1. Introduction

As an important part of the multi-level medical security system in China, commercial health insurance has developed rapidly with the strong support of policies. (In China, the multi-level medical security system is divided into three parts. Among them, basic medical insurance is the main body, medical assistance is the backing, and supplementary insurance, mainly commercial health insurance is the extension). During the 13th Five-Year Plan period, more than 1.6 billion new health insurance policies were issued, with a total insurance amount of nearly 300 trillion yuan, and 250 million people were paid health insurance compensation. China’s health insurance premium income reached RMB 844.7 billion in 2021, achieving a positive growth of 3.2% against the background of negative growth of the insurance industry hit by COVID-19. In this context, the development of commercial health insurance has also taken on new features. Commercial health insurance is an insurance in which commercial insurance institutions pay compensation for losses caused by health reasons and medical behaviors. First, the market acceptance of commercial health insurance is constantly improving. With the improvement of living standards, people’s health awareness and risk awareness are gradually improving. The outbreak of COVID-19 has further stimulated people’s demand for commercial health insurance, and the market’s attention and recognition of commercial health insurance has reached a new height. Secondly, commercial health insurance has gradually formed a new trend of integrated development in multiple fields. In the process of continuous innovation and development, the insurance industry has expanded along the health industry chain to medical treatment, medicine, health services and other industries. The application of new technologies such as the internet, big data and artificial intelligence further promotes the integrated development of health insurance and the allocation and optimization of medical and health resources [1]. Finally, the positive externality effect of commercial health insurance has been significantly improved. Studies have shown that health insurance plays an increasingly important and positive role in improving health levels, stimulating economic growth and promoting social progress [2,3]. In general, the role of commercial health insurance is not limited to the insurance market itself, and its important role in improving the medical security system, promoting industry integration and even promoting economic efficiency has also attracted increasing attention.

Against this background, it is of great significance to analyze the specific role of commercial health insurance in medical security, economy and other fields, and evaluate the specific path of commercial health insurance to promote economic efficiency, which is to guide the long-term development of China’s commercial health insurance. Based on this, this study firstly analyzes the action mechanism of various channels in the process of promoting the economic efficiency of commercial health insurance, makes it clear that commercial health insurance does have a promotion effect on economic efficiency, and constructs an effective path for the development of commercial health insurance to promote economic efficiency. Finally, the empirical method is used to test this process.

The contents of this study are arranged as follows: in the second part, the specific mechanism of promoting economic efficiency of commercial health insurance is discussed theoretically, and the practical path of exerting the economic efficiency of commercial health insurance is summarized. The third part constructs the commercial health insurance development index and economic efficiency index, and proposes the model hypothesis. The fourth part selects the relevant data of 31 provinces and regions in China from 2007 to 2019 for empirical analysis to evaluate the role of commercial health insurance in improving economic efficiency. The fifth section presents the conclusion.

## 2. Mechanism of Commercial Health Insurance to Improve Economic Efficiency

By playing a role of “self-organization” in the market economy [4], commercial health insurance first helps to reduce the financial vulnerability of households, release precautionary savings of residents, and improve overall health levels by guiding residents’ behaviors. In addition, as an important node of the health industry chain, commercial health insurance promotes the integrated development of the health industry with more flexible market arrangements, efficiently integrates and utilizes market resources, and promotes the rationalization of medical expenses. As an important part of the financial service industry, commercial health insurance collects funds and creates added value with more professional risk management and fund management means, thus promoting the accumulation of all kinds of capital and promoting the growth of national income. In summary, compared with public medical security plans, market-oriented commercial health insurance regulates the relationship between supply and demand on the basis of voluntariness and profit, and has significant advantages in professional operation, management efficiency, innovation ability and other aspects [4]. Therefore, it can be used as an effective supplement to social medical insurance. Through product and service innovation, commercial health insurance can smooth the positive development cycle and improve economic efficiency.

### 2.1. Give Full Play to the Market-Oriented Risk Protection Function

Reduce household financial vulnerability. While actively participating in the socialized operation of basic medical insurance, commercial health insurance provides a strong supplement to individual and family health insurance plans.

By providing diversified products and services for the treatment of diseases, medical management and nursing care, commercial health insurance plays the role of economic compensation and risk transfer, alleviating the economic pressure on individuals or families caused by large expenditures exceeding the reimbursement limit, and effectively reducing the vulnerability of families to risks [5,6,7].

Release household precautionary savings. For individuals, commercial health insurance with accurate strategic positioning has a smoothing effect, which can solve the balance problem of health demands in the whole life cycle and further meet residents’ demands for wealth protection at a higher level [8]. In addition, commercial health insurance will adjust the consumption and savings structure of residents in the process of health risk transfer and management. While improving the consumption level, the inefficient individual precautionary savings should be transformed into efficient centralized savings, and the effective allocation of funds across sectors should be realized through the investment management function of commercial health insurance companies [3]. At the same time, the precautionary savings released by having commercial health insurance can enhance investment incentives for poor and vulnerable households, improve the efficiency of residents’ capital utilization and the growth rate of individual income [9].

### 2.2. Promoting Targeted Risk Reduction Management

Improve personal health status. First of all, commercial health insurance cooperates with medical, physical examination, nursing and other institutions to establish a professional health management platform, actively intervene and guide customers’ health behaviors, and provide customers with full-cycle health protection. Secondly, commercial health insurance provides a wide range of products and services to policyholders, providing comprehensive medical support and health risk management services in disease prevention, medical services, infertility, nursing and other fields [8]. Finally, commercial health insurance provides convenient medical services for the insured in the whole process through designated hospitals, off-site medical services, medical networks and green channels. In general, commercial health insurance can improve the national health level through the protection and extension services. It can effectively reduce the social medical burden and maximize social efficiency [1]. Furthermore, it can promote the accumulation of family human and physical capital [10], effectively drive health investment and improve the health status of the insured individuals, thus promoting the overall quality of the labor force in society [11].

Optimize the allocation of medical resources. A series of studies represented by the RAND Health Insurance Experiment show that the widespread moral hazard in the health insurance market is the main reason for the unreasonable medical expenditure rise of the social basic medical insurance. Compared with social medical insurance, commercial health insurance institutions can establish a check and balance mechanism between the insured and medical institutions with more flexible payment methods and cooperation modes, urge medical institutions to control medical costs from the supplier side, and reduce excessive consumption of medical resources [12]. Moreover, there is extensive competitive pressure in the commercial health insurance market, and insurance institutions have more sufficient incentive mechanisms to reduce costs, thus playing a greater role in promoting the rationalization of medical expenses. In addition, in reality, commercial health insurance companies can also take advantage of their efficiency in market-oriented operation and professional risk management to assist government departments in managing social insurance.

Promote the coordinated development of the health industry. Commercial health insurance not only plays a supplementary role to social medical insurance, but also plays an important role in promoting the construction of the health service system [4]. First of all, commercial health insurance can improve the utilization of medical and health resources. Commercial health insurance involves various fields such as medical services, medicine supply, and health management. It is the core of the health industry chain and an important provider of funds [13]. First, it can reduce the cost of resource transfer across departments and guide the orderly flow of resources to improve the resource utilization efficiency of the health industry chain. Moreover, integrating with medical care, physical examination, nursing care, and drug distribution companies can give full play to the technical advantages of insurance companies in risk management and the institutional advantages of capital utilization [1]. Second, commercial health insurance can improve the service efficiency of medical institutions. Commercial insurance companies use various methods to establish cooperative relationships with medical institutions and pharmaceutical groups, innovate and improve product services, and improve medical risk management systems. Therefore, commercial health insurance can jointly build a data platform for insurance and medical treatment from the level of information circulation, effectively improving the service efficiency of medical institutions [14]. Finally, commercial health insurance can promote the innovation and development of the health industry. With the gradual expansion of the commercial health insurance market, commercial health insurance products provide a guarantee for the innovation and development of medicine and medical technology, and can also provide financial support for the innovation of scientific research institutions and pharmaceutical companies, promote the research and development and application of new technologies, new drugs, and new medical devices, and further promote the innovative development of the health industry [15]. For example, in March 2020, Ping An Life Insurance, a subsidiary of Ping An of China, invested in Shionogi Pharmaceutical and became its important strategic shareholder. Recently, Shionogi Pharmaceutical submitted the approval for the manufacture and sale of oral drugs for the new coronavirus to the Ministry of Health, Labor and Welfare (MHLW) of Japan.

### 2.3. Promoting Capital Accumulation and Financing

Promote capital accumulation. Commercial health insurance companies have accumulated a large amount of prepaid insurance premiums in the process of risk management. Through asset management activities, such as investing in bonds, securities investment funds, and interbank lending, they play an important role in promoting gross domestic product (GDP) growth, increasing national tax revenue, and stabilizing the financial system [16,17]. Moreover, the operation of commercial health insurance focuses on using data as a new factor of production, analyzing and applying it through InsurTech, realizing the transfer of data from unstructured to structured, and improving the risk selection, underwriting structure, and pricing capabilities of the traditional insurance industry. (The International Association of Insurance Supervisors (IAIS) first defined InsurTech in March 2017 as a branch of FinTech in the insurance field, which is “the sum of all kinds of emerging technologies and innovative business models that have the potential to change the insurance business”). Finally, it can alleviate the adverse selection and moral hazard problems caused by information asymmetry, and realize the efficient accumulation and use of data capital [18].

Alleviate government financial pressure. Commercial health insurance under the market mechanism can effectively alleviate the government’s financial pressure through its unique financing function: First, commercial health insurance can allow the government to invest resources in low-income, high-risk and other socially vulnerable groups, thereby improving resource utilization efficiency [19]. Second, transferring rigid inputs, such as equipment, systems, and personnel to commercial insurance companies, commercial health insurance can significantly reduce government spending on medical security, further improve the economic efficiency of government fund utilization, and promote social stability and development.

## 3. China’s Commercial Health Insurance Development and Economic Efficiency Evaluation

In order to comprehensively evaluate the economic efficiency brought by China’s commercial health insurance in the process of operation, this part first constructs the commercial health insurance development index, and then constructs the economic efficiency index from the three paths of commercial health insurance to promote economic efficiency.

### 3.1. Measure the Development Level of Commercial Health Insurance

Starting from the three dimensions of efficiency, fairness and sustainability, this study selects five indicators including: the depth of health insurance, the density of health insurance, the ratio of special business premium income to the regional population, the proportion of the total compensation of commercial health insurance to the total medical expenses, and the ratio of long-term health insurance liability reserves to total assets, to construct the Commercial Health Insurance Development Index (CHIDI). The specific ideas are as follows:

1. Development Efficiency. The development of economic efficiency is the unity of quality and quantity, and its existence must be based on a certain quantity, which requires the continuous relative balance of element supply to achieve efficient resource allocation [20]. For the insurance industry, the core manifestation of development efficiency is the commercial health insurance depth representing the ratio of commercial health insurance premiums to local GDP, and the commercial health insurance density of per capita commercial health insurance premiums in the region. These two indicators can respectively reflect the degree of penetration of premium income into economic development in a region and the popularity of the insurance industry in the region. Therefore, it can measure the operating efficiency of the regional health insurance market [21], and can be used as a reasonable measure of the development efficiency of commercial health insurance.

2. Development Fairness. Combined with the research results of Yang and Jiang (2018) [22], this study focuses on the ratio of the premium income of the entrusted management business of basic health security and the special business of health insurance to the population of the region in different regions. The fairness of measuring economic efficiency can be examined from the perspective of the universality of health insurance supply. Among them, the special business premium income mainly focuses on the level of social security provided by health insurance. This indicator reflects the inclusive protection effect of commercial health insurance beyond the arrangement of the market mechanism, and reflects the effect of commercial health insurance on social welfare.

3. Development Sustainability. This study comprehensively evaluates the sustainability of commercial health insurance in two respects, which are risk protection capability and business sustainability. Risk protection capability emphasizes the extent to which commercial health insurance can respond to policy calls, improve the level of protection, and protect the interests of consumers under the guidance of returning to the origin of insurance. In this study, the ratio of the total compensation amount of commercial health insurance to the total medical expenses is selected as the proxy variable of the indicator of risk protection capability. In terms of business sustainability, it is necessary to examine the stability of the development of insurance companies. The operation of long-term insurance not only provides stable and predictable protection for policyholders, but also urges the industry to pursue more accurate risk pricing, better product design, and more stable operating style. Therefore, this study uses the ratio of long-term health insurance liability reserve to total assets to measure the degree of business sustainability of the insurance industry.

**Figure 1:**
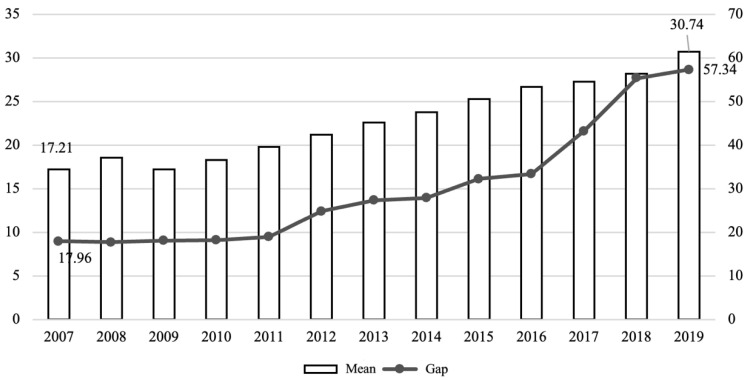
Mean and gap of commercial health insurance development index in various regions from 2007 to 2019.

The specific index settings are shown in Table 1:

For each of the selected indicators, firstly, dimensionless processing is performed, and the data interval is converted into a score of 0–100. Then, the specific indicator scores are weighted by the entropy method to obtain the final score of the evaluation object, that is, each provincial commercial health insurance development index (CHIDI) for 2007–2019. The index weight value of the entropy weight method is obtained based on the amount of information reflected by the variation degree of each measurement index data, which reduces the interference of subjective and human factors during index weighting [23,24]. The specific process is: first construct the initial indicator matrix, then calculate the contribution of different indicators in each region during the sample period, then calculate the entropy value of the indicator to obtain information entropy, and finally obtain the weight of each indicator. In terms of the definition of CHIDI in this study, the final numerical score should be in the range of 0–100, and the larger the value, the higher the development level of commercial health insurance in the region. According to the steps of constructing the index, this study calculates the commercial health insurance development index of 31 provinces, municipalities, and autonomous regions in China. The average level of health insurance development in each region from 2007 to 2019 is shown in Figure 1. It can be seen that the average development level of commercial health insurance in each region has gradually increased in recent years, and the largest gap between different regions in the same year also showed an expanding trend (that is, the highest number of the health insurance development index minus the lowest number of the health insurance development index in that year, expressed as “gap”). In 2007, the difference between the maximum and minimum values of CHIDI was only 30 points, while the gap reached its highest point in 2016 when it was 78 points. Specifically, there are big differences between regions in the past 13 years. The highest level of development is Beijing, with an average value of 55.56 over the years, and the lowest is Tibet, with an average value of only 6.38 during the investigation period.

The reason for the regional imbalance and widening gap in commercial health insurance is rooted in the behavioral pattern of health insurance demand and the cumulative causal phenomenon of economic and social development [21]. This may also imply that commercial health insurance has not developed simultaneously with the expansion and quality improvement of the social medical insurance system. Due to the late start and short time of commercial health insurance, the market generally has problems, such as single product structure, serious homogeneity, and insufficient innovation, which largely restrict the transformation of commercial health insurance purchase intentions into actual purchases.

### 3.2. Quantitative Measurement of Commercial Health Insurance Promoting Economic Efficiency

According to the analysis of the theoretical mechanism, commercial health insurance can firstly affect the basic indicators of economic development through the risk protection function; secondly, it can promote the social efficiency of economic development through risk reduction management; finally, it can bring about industrial changes in economic development through the improvement of financial intermediation functions. Ultimately, it acts on the improvement of economic efficiency. Based on this, drawing on the compilation experience of Shi and Zhang (2019) [25], this study compiles the Economic Efficiency Index (EEI) from three perspectives: the basis of economic development, the social efficiency of economic development, and the industry transformation brought about by economic development. The specific impact mechanism of commercial health insurance development promoting economic efficiency growth can be characterized as:

$$\begin{array}{l} \mathrm{Economic}\;\mathrm{Efficiency}\\ \;\;\;\;\;\;\;\;\;\;\;\;\;\;\;\;\;\;\;\;\;\;\;\;=\delta \cdot \mathrm{Development}\;\mathrm{of}\;\mathrm{Economic}\;\mathrm{Basis}+\eta \\ \;\;\;\;\;\;\;\;\;\;\;\;\;\;\;\;\;\;\;\;\;\;\;\;\cdot \mathrm{Development}\;\mathrm{of}\;\mathrm{Social}\;\mathrm{Efficiency}+\varphi \\ \;\;\;\;\;\;\;\;\;\;\;\;\;\;\;\;\;\;\;\;\;\;\;\;\cdot \mathrm{Development}\;\mathrm{of}\;\mathrm{Industry}\;\mathrm{Transformation} \end{array}$$

$$\mathrm{Development}\;\mathrm{of}\;\mathrm{Economic}\;\mathrm{Foudamentals}=\alpha_{1}\cdot \mathrm{Intensity}+\alpha_{2}\cdot \mathrm{Stability}+\alpha_{3}\cdot \mathrm{Openness}$$

$$\mathrm{Development}\;\mathrm{of}\;\mathrm{Social}\;\mathrm{Efficiency}=\beta_{1}\cdot \mathrm{Income}+\beta_{2}\cdot \mathrm{Employment}+\beta_{3}\cdot \mathrm{Education}$$

$$\begin{array}{l} \mathrm{Development}\;\mathrm{of}\;\mathrm{Industry}\;\mathrm{Transformation}\\ \;\;\;\;\;\;\;\;\;\;\;\;\;\;\;\;\;\;\;\;\;\;\;\;\;\;\;\;\;\;=\gamma_{1}\cdot \mathrm{Development}\;\mathrm{of}\;\mathrm{the}\;\mathrm{medical}\;\mathrm{industry}+\gamma_{2}\cdot \mathrm{Development}\;\mathrm{of}\;\mathrm{medical}\;\mathrm{insurance}\;\mathrm{fund} \end{array}$$

The specific construction ideas and corresponding model assumptions of the indicator system are as follows:

1. Development of Economic Basis. This study divides the development of an economic basis into three dimensions: economic development intensity, stability, and openness: (1) The intensity of economic development. It represents the economic growth vitality of a region. The higher the level, the more prosperous the economic development of the region, the higher the living standard and quality of life of the residents, and the more abundant the momentum of economic development [25], represented by the gross domestic product (GDP). (2) The stability of economic development. It represents the sustainability of a region’s development, represented by the reciprocal of the coefficient of variation of the economic growth rate. Based on the three-year rolling window, this study calculates the coefficient of variation of the economic growth rate of each region. The higher the coefficient of variation, the more unstable the economic growth rate of the region, and it is difficult to guarantee the sustainability of its economic development. (3) The openness of economic development. An open economy helps to improve total factor productivity through learning by doing, technology spillover, and attracting foreign direct investments (FDI). It is represented by the total investment amount of foreign-invested enterprises at the end of each year by region. According to theoretical analysis, the economic compensation and risk transfer functions of commercial health insurance can reduce the vulnerability of household finances, effectively alleviate the burden of government fiscal expenditures, and release residents’ precautionary savings. Therefore, commercial health insurance can improve the consumption level of residents and the liquidity of personal and family funds, and generally promote the sustained growth of basic economic indicators. Based on this, the research hypothesis H1a of this study is proposed:

**H1a:**  *The development of commercial health insurance can promote the development of the economic basis*.

2. Development of Social Efficiency. Referring to the compilation experience of Li and Ren (2020) [26], the welfare changes and distribution of benefits of economic growth are reflected in the emergence of people-centered social efficiency, which can be quantified in three respects: income, education and employment levels. (1) Consider the distribution of economic results and the allocation of output at the micro level, that is, the distribution of residents’ income. Under a certain output level, an excessive income distribution gap among residents will reduce the overall welfare level of the society, thereby affecting the quality of economic development [27]. Therefore, the variable representative of residents’ disposable income, which directly reflects wealth accumulation and financial status, is selected. (2) Education is the specific embodiment of human capital from the perspective of knowledge and innovation, and is a key factor for the sustainability and long-term development of China’s high-quality economic development. It is represented by the ratio of the number of students in general undergraduate colleges and universities in the area to the number of permanent residents at the end of the year [23]. (3) Employment measures the fluctuation level of human capital, which directly affects the welfare of residents. Therefore, this study uses the unemployment rate to measure the direct impact of economic fluctuations on residents’ welfare. According to theoretical analysis, residents can reduce the vulnerability of their families by purchasing commercial health insurance, and also reduce the cost of serious diseases and reduce the risk of returning to poverty due to illness, and the risk of poverty due to illness; thus, the family’s disposable income increases significantly. Furthermore, education and employment opportunities for families will also increase, which will increase the accumulation of human capital and fully share the benefits of economic development. Based on this, the research hypothesis H1b of this study is proposed:

**H1b:**  *The development of commercial health insurance can promote the development of social efficiency*.

3. Development of Industry Transformation. This study takes into account the medical security attributes of commercial health insurance, and measures the industrial transformation of economic efficiency from the perspective of the role of commercial health insurance in terms of medical service utilization and medical insurance fund pressure relief. (1) Development of the medical industry, using the number of health technicians per 10,000 people as a proxy variable, reflects the sharing level of medical service utilization. It embodies the logical approach of bringing about shared development brought about by industry changes and promoting the realization of economic efficiency growth [28]. (2) Development of medical insurance funds, using per capita medical insurance expenditure as a proxy variable [29]. According to theoretical analysis, commercial health insurance promotes the industry transformation in two respects: On the one hand, commercial health insurance alleviates the pressure on medical insurance fund expenditures and optimizes the allocation of medical insurance funds through professional management and technical means. On the other hand, the protective role of commercial health insurance has driven residents to pay more attention to their own health conditions, and the degree of utilization of medical institution services has increased. Based on this, the research hypothesis H1c of this study is proposed:

**H1c:**  *The development of commercial health insurance can promote the development of industry transformation*.

Based on the above effects, this study further proposes the research hypothesis H2:

**H2:**  *The development of commercial health insurance can promote the growth of economic efficiency*.

**Figure 2:**
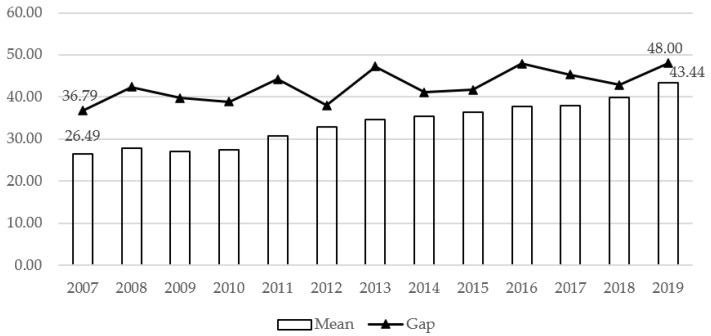
EEI of each region from 2007 to 2019. Note: The above figure is the estimated data, and the original data comes from the China Statistical Yearbook over the years.

**Figure 3:**
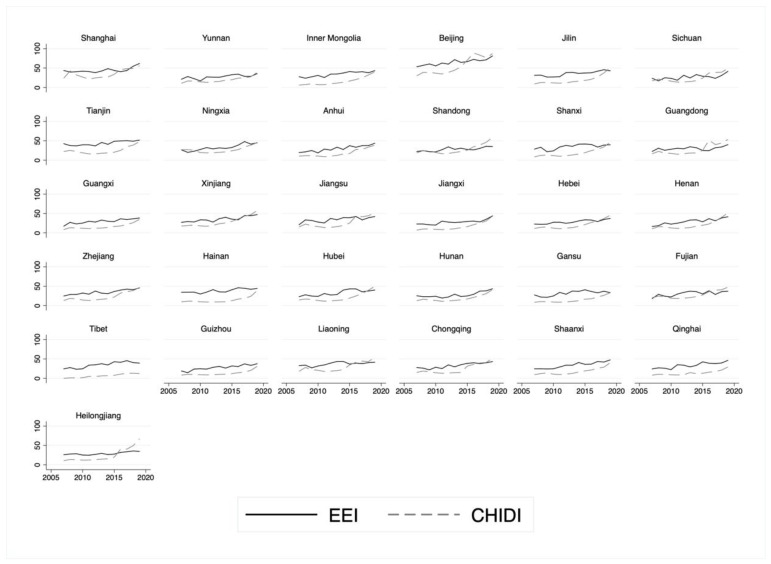
Relationship between EEI and CHIDI by region.

In the construction of specific indicators, due to the heterogeneity of various indicators of the development of the economic basis, social efficiency and industry transformation, it is necessary to convert the original data of each indicator into dimensionless indicator measurement values through standardized methods. In order to ensure the comparability and objectivity of the measurement results, this study follows the practice of Shi and Zhang (2019) [25], using the equal weight method to assign values, and assigning 1/n weights to the secondary indicators under the three first-level indicators (n is the number of second-level indicators), and then the first-level indicators are given an equal weight of 1/3 to express the concept of comprehensive and coordinated development in the process of improving economic efficiency. Figure 2 shows the change of economic efficiency index (EEI) in different regions from 2007 to 2019. It can be seen that the level of economic efficiency has gradually increased in recent years, while the largest gap between different regions in the same year (that is, the number with the highest efficiency index in the year minus the lowest number of the index in the past year, denoted as “gap” in the figure) is also showing a trend of widening. The difference between the maximum value and the minimum value in 2007 was only 36, while the gap reached its highest in 2019, which was 48. In the past 13 years, the economic efficiency of various regions has been quite different. The highest level is in Beijing, with an average value of 65.13 over the years, and the lowest level is in Sichuan, with an average value of only 26.86 in 13 years. This phenomenon is basically consistent with the Commercial Health Insurance Development Index (CHIDI) (See Figure 3). Numerically speaking, there is a certain relationship between the development of commercial health insurance and economic efficiency. However, whether this relationship is significant and robust needs to be tested empirically.

## 4. Empirical Analysis

### 4.1. Research Design

#### 4.1.1. Data Selection and Descriptive Statistics

In order to quantify the impact of commercial health insurance on the growth process of economic efficiency, this study uses the method of empirical analysis to investigate the situation of 31 provinces, municipalities and autonomous regions in China. The explained variable used in the empirical analysis is EEI; the core explanatory variable is CHIDI.

In terms of control variables, drawing on the experience of previous studies and avoiding the influence of multicollinearity, this study selects the following variables: (1) The level of urbanization. Studies by Schnabl (2008) [30] etc. pointed out that the level of urbanization significantly affects the improvement of regional economic efficiency, which is represented by the ratio of urban population of each province at the end of the year to the total population of each province. (2) Aging level. Studies have shown that the acceleration of aging is likely to have a negative impact on economic growth [31], and the health problems brought about by an aging population have become an important challenge to economic and social development [15], which is closely related to the function of commercial health insurance discussed in this study. We use the proportion of the local population aged 65 and over. (3) Local financial expenditure. Fiscal expenditure is one of the main tools of fiscal policy, and it has an important influence on maintaining stable economic development and improving economic quality. This study uses the total annual local fiscal expenditure. (4) Local industrial structure. A reasonable local industrial structure significantly contributes to the improvement of the quality of economic development [32], which is represented by the ratio of the added value of the tertiary industry to the regional GDP in this study. (5) Infrastructure construction. Infrastructure construction provides a basic guarantee for the promotion of cross-regional material and personnel flows in the process of economic development, reduces the friction loss of various production factors, and ultimately provides a guarantee for the realization of better economic development results. In this study, the total mileage of roads and railways per square kilometer is expressed.

The data used in this study come from the 2007–2020 China Statistical Yearbook, China Insurance Yearbook, China Health Statistical Yearbook, China City Statistical Yearbook and the official website database of the National Bureau of Statistics. Brief descriptive statistics of the main variables and other control variables used to construct the CHIDI and EEI are shown in Table 2.

#### 4.1.2. Model Setting

Based on theoretical analysis, in order to investigate the role of commercial health insurance in promoting economic efficiency, this study establishes the following regression model:

(1) $$EEI_{it}=\beta_{0}+\beta_{1}CHIDI_{it}+\beta_{2}Control_{it}+\lambda_{i}+\eta_{t}+\varepsilon_{it}$$

Among them, $\eta_{t}EEI_{it}$ represents the economic efficiency level of region *i* in year *t*. $CHIDI_{it}$ represents the development index of commercial health insurance in region *i* in year *t*. $Control_{it}$ is the control variables, including urbanization level, aging level, local fiscal expenditure, local industrial structure and infrastructure construction. $\lambda_{i}$ represents regional fixed effects, $\eta_{t}$ represents year fixed effects, and $\varepsilon_{it}$ is a random disturbance item.

In order to further test the specific path of commercial health insurance to promote economic efficiency growth, the three-dimensional indicators for measuring economic efficiency are also included in the regression model:

(2) $$index_{it}=\beta_{0}+\beta_{1}CHIDI_{it}+\beta_{2}Control_{it}+\lambda_{i}+\eta_{t}+\varepsilon_{it}$$

Among them, $index_{it}$ represent the development of economic basis, social efficiency and industry transformation, and other variables are the same as in model (1).

### 4.2. Empirical Analysis

#### 4.2.1. Baseline Regression

Based on the panel data of 31 provinces, municipalities and autonomous regions in China from 2007 to 2019, the fixed effect model was used and the three dimensions of measuring economic efficiency growth were introduced into the model for investigation. The results are shown in Table 3. The regression results support the hypothesis of this study that the development level of commercial health insurance has a significant positive effect on the process of economic efficiency growth. The three dimensions constructed in this study to measure economic efficiency growth are significantly affected by the development of commercial health insurance. Moreover, the development of commercial health insurance has significantly promoted the improvement of economic efficiency.

#### 4.2.2. Robustness Tests

Substitute core explanatory variables. In order to examine the robustness of the established model and the reliability of the index constructed in this study, commercial health insurance depth and density were used to replace the core explanatory variable CHIDI for testing. The regression results are shown in Table 4, which are basically consistent with the baseline regression results.

Delete specific samples. The level of health insurance in municipalities has developed rapidly, and economic efficiency is often high, so the problem of reverse causation may be serious. Referring to the research experience of Luo et al. (2016) [33], this study excludes the samples of municipalities, and the coefficient of CHIDI is still significantly positive.

Heterogeneity test. Considering the imbalance in regional development, in order to further observe the impact of commercial health insurance development in different regions on economic efficiency growth, we divide the sample into three sub-samples in the eastern region, middle region and western region. The CHIDI was used as the explanatory variable for fixed effect regression, and the results are shown in Table 5. The regression results are basically consistent with the results of the full sample, which once again confirms the robustness of the conclusions investigated in this study. Commercial health insurance can significantly promote the improvement of economic efficiency, but the degree of significance varies in different regions. Relatively speaking, the more economically developed the region, the more obvious the role of commercial health insurance is; as shown in the table, the significant level of the eastern region is higher than that of the middle and western regions. This shows that the impact of commercial health insurance on economic efficiency is also restricted by the economic environment itself, and the more developed the economy, the more obvious this effect will be.

#### 4.2.3. Endogeneity Test

Because residents in areas with higher economic efficiency may have a clearer understanding of insurance and have a stronger willingness to purchase commercial health insurance to protect their own health, there may be an endogenous bias caused by reverse causality between the development level of commercial health insurance and the level of regional economic efficiency. In order to solve this problem, this study refers to the research experience of Chong et al. (2013) [34]. The mean value of the average commercial health insurance development index in the bordering regions of the investigated regions is used as an instrumental variable for the development of commercial health insurance in the region. In terms of correlation, studies have confirmed that there is a spatial spillover effect in the development of commercial health insurance [35], and commercial health insurance in various regions presents a trend of mutual promotion and common development. Therefore, the development of commercial health insurance in bordering areas and the development of commercial health insurance in target areas must have a high degree of correlation. In terms of exogenousness, the development level of commercial health insurance in similar regions is often limited to the region, and the impact on itself is still in its infancy, so it is even more difficult to affect the economic efficiency of the development of the target region. Column (1) in Table 6 reports that in the regression with CHIDI as the explanatory variable in the first stage, the selected instrument variable (IV) is significantly positively correlated with CHIDI, which confirms the conclusions of previous studies that there is a spatial spillover effect in the development of commercial health insurance. In the second stage, in order to ensure the reliability of IV and avoid the problem of weak IV, the limited information maximum likelihood method (LIML), which is less sensitive to weak IV, is used. The second-stage regression results show that the coefficient of CHIDI is still significantly positive at the 1% level, indicating that after mitigating the endogenous problems that may exist in the model, the conclusion of this study still holds true. The development of regional commercial health insurance can significantly promote the improvement of local economic efficiency.

There is likely to be a serial correlation in the economic efficiency level of the region. We further use the systematic generalized method of moments (GMM) regression to alleviate the endogeneity problem and test the robustness of the conclusion. The EEI with a lag of two periods is introduced into the model, and the test statistics show that IV is effective, which satisfies the use conditions of the system GMM. We also simultaneously introduce the IV selected above into the GMM. The results are reported in the third column of Table 7. The regression results show that the coefficient of CHIDI is significantly positive at the 5% level in the regression. This shows that after controlling the level of lagging economic efficiency and the endogenous problems it causes, the promotion effect of the development of commercial health insurance on economic efficiency still exists, and the previous conclusion is robust.

## 5. Conclusions

From the perspective of the construction of China’s medical security system, commercial health insurance, as an important value enabler of basic medical insurance supplementary protection, can promote the rational use of medical resources, control medical costs, and provide patients with better medical care through market and technical means. In this way, commercial health insurance can bring economic efficiency to society. In this context, it is very important to have a deep understanding of the mechanism of commercial health insurance in promoting economic efficiency. Based on this understanding, this study conducts an empirical analysis of the relationship between the development of commercial health insurance and economic efficiency in 31 provinces, municipalities and autonomous regions in China from 2007 to 2019, and finds that the development of commercial health insurance can significantly positively promote the growth of economic efficiency. Also, it plays an important role in promoting the development of the economic basis, social efficiency and industry transformation. The regression results of the robustness test and endogeneity test also show that the positive effect of commercial health insurance on economic efficiency is widespread.

Therefore, we believe that further promoting the positive feedback effect between commercial health insurance and economic development requires a correct understanding of the functions and efficiency of commercial health insurance, and be confidence in the value of the industry. Through this study we find that commercial health insurance can promote economic efficiency and play the role of value creator. In this sense, in the process of improving China’s multi-level medical security system, efforts should be made to build a new medical security model in which commercial health insurance and social medical insurance complement, cooperate, and develop with each other, and develop together. The important role of commercial health insurance cannot be ignored just because the market size of commercial health insurance in China is still small.

## Figures and Tables

**Table 1:** Commercial Health Insurance Development Index (CHIDI).

Dimension	Indicators	Description	Data Resources
Efficiency	Health insurance depth	Ratio of commercial health insurance premiums to local GDP	CBIRC
	Health insurance density	Regional per capita commercial health insurance premiums	CBIRC
Fairness	Density of special business insurance premium income	Ratio of special business insurance premium income to regional population	CBIRC
Sustainability	Proportion of commercial health insurance compensation	Ratio of the total compensation amount of commercial health insurance to the total medical expenses	CBIRC& China Health Statistical Yearbook
	Proportion of commercial health insurance liability reserve	Ratio of long-term health insurance liability reserves to total assets	CBIRC

**Table 2:** Simple descriptive statistics.

	Mean	Med.	S.D.	Min	Max	Obs
EEI	33.696	33.286	9.5324	14.507	81.435	403
CHIDI	22.452	18.128	13.972	0.0387	88.852	403
Aging level	9.8375	9.6356	2.2051	4.8630	16.265	403
Local fiscal expenditure	3830.5	3312.2	2675.4	241.85	17298	403
Urbanization	54.285	5.3205	1.4096	21.453	89.607	403
The added value of the tertiary industry	9328.4	6227.6	9415.7	196.60	60268	403
Mileage of first-class highway	0.2632	0.1400	0.3043	0.0000	1.5300	403
Road mileage	13.959	14.320	7.5431	1.1200	33.710	403
Local industrial structure	47.086	46.219	9.2534	29.792	83.688	403
Infrastructure	2.1536	1.2520	2.0508	0.0000	9.5685	403
GDP per capita	43512	37580	25772	7778	161776	403
Average local total foreign investment (million yuan)	65.545	56.825	38.832	10.181	287.30	403
Gini coefficient	0.4274	0.4258	0.0449	0.3502	0.5097	403
Number of students enrolled in primary schools	323.00	261.40	242.23	29.200	1092.9	403
Unemployment rate	3.3838	3.5000	0.6511	1.2000	4.6000	403
Per capita medical insurance expenditure	1268.4	1089.4	741.40	292.42	5340.0	403
Long-term health insurance liability reserve ratio to total assets	5.0995	4.7172	1.9883	0.0000	10.793	403
Commercial health insurance premium income to total medical expenditure	19.519	14.884	14.444	0.0583	81.216	403
The ratio of the premium income of the eight major businesses to the resident population	13.875	7.0490	19.290	0.0000	124.99	403
Commercial Health Insurance Density	170.98	83.876	224.04	0.3460	1861.7	403
Commercial Health Insurance Depth	0.3124	0.2196	0.2389	0.0000	1.5135	403

**Table 3:** Baseline Regression.

	Fundamentals	Social Efficiency	Industry Transformation	EEI
CHIDI	0.135 *** (3.882)	0.151 * (1.691)	0.303 *** (7.433)	0.106 *** (2.826)
Aging level	0.023 (1.480)	−0.005 (−0.134)	−0.028 (−1.151)	−0.003 (−0.192)
Urbanization level	0.189 *** (8.499)	0.004 (−0.070)	0.118 *** (4.530)	0.101 *** (4.221)
Local fiscal expenditure	−0.358 *** (−15.73)	0.075 (1.299)	−0.030 (−1.143)	−0.104 *** (−4.270)
Infrastructure	0.004 (0.020)	0.928 *** (2.042)	0.176 ** (0.856)	0.369 * (1.931)
Local industrial structure	0.208 *** (5.756)	0.577 * (1.694)	0.679 *** (16.07)	0.489 *** (12.56)
Place FE	Y	Y	Y	Y
Year FE	Y	Y	Y	Y
Adjusted *R*^2^	0.749	0.282	0.812	0.736
Obs	403	403	403	403
*F*-value	64.00	9.32	92.62	59.98
*p*-value	0.000	0.000	0.000	0.000

Notes: * *p* < 0.01, ** *p* < 0.05, *** *p* < 0.01. In the above table, the corresponding t statistics are in parentheses.

**Table 4:** Robustness Tests.

	EEI		
CHI Depth	0.134 * (3.220)		
CHI Density		0.099 *** (4.610)	
CHIDI			0.128 *** (2.885)
Controls	Y	Y	Y
Place FE	Y	Y	Y
Year FE	Y	Y	Y
Adjusted *R*^2^	0.738	0.704	0.618
Obs	403	403	351
*F*-value	60.47	137.3	30.74
*p*-value	0.000	0.000	0.000

Notes: * *p* < 0.01, *** *p* < 0.01. In the above table, the corresponding t statistics are in parentheses.

**Table 5:** Heterogeneity Test.

	Eastern Region	Middle Region	Western Region
CHIDI	0.455 *** (11.15)	0.164 * (1.681)	0.070 (1.215)
Controls	Y	Y	Y
Place FE	Y	Y	Y
Year FE	Y	Y	Y
Adjusted *R*^2^	0.846	0.684	0.701
Obs	169	78	156
*F*-value	124.3	82.93	20.10
*p*-value	0.000	0.000	0.000

Notes: * *p* < 0.01, *** *p* < 0.01. In the above table, the corresponding t statistics are in parentheses.

**Table 6:** IV Regression.

	First Stage	Second Stage	
		2SLS	LIML
Variables	CHIDI	EEI	
IV_AVG_CHIDI	0.684 *** (11.33)		
CHIDI		0.164 *** (3.246)	0.164 *** (3.246)
Controls	Y	Y	Y
Place FE	Y	Y	Y
Year FE	Y	Y	Y
Obs	403	403	403

Notes: *** *p* < 0.01. In the above table, the corresponding t statistics are in parentheses.

**Table 7:** System GMM Regression.

	EEI	
l. EEI(1)	0.394 *** (4.711)	0.394 *** (5.530)
l. EEI(2)	0.133 (1.570)	0.149 * (1.682)
CHIDI	0.072 ** (2.452)	0.088 ** (2.450)
Controls	Y	Y
AR(2)	0.282 (0.778)	0.166 (0.868)
Hansen	18.68 (0.720)	24.31 (0.690)
Place FE	Y	Y
Year FE	Y	Y
Obs	341	341

Note: (1) * *p* < 0.01, ** *p* < 0.05, *** *p* < 0.01. In the above table, the corresponding t statistics are in parentheses. (2) AR(2) indicates the second-order sequence autocorrelation test, and the *p* value is in brackets. (3) Hansen indicates an overidentification test for dynamic panel instrumental variables, with *p* values in parentheses.

## Data Availability

Restrictions apply to the availability of these data. Data was obtained from China Banking and Insurance Regulatory Commission (CBIRC) and are available from the authors with the permission of CBIRC.
